# Contributions of Farm Animals to Immunology

**DOI:** 10.3389/fvets.2018.00307

**Published:** 2018-12-06

**Authors:** Efrain Guzman, Maria Montoya

**Affiliations:** ^1^The Pirbright Institute, Woking, United Kingdom; ^2^Centro de Investigaciones Biológicas, CIB-CSIC, Madrid, Spain

**Keywords:** comparative immunology, vaccines, dendritic cells, bovine immunology, porcine immunology, chicken immunology

## Abstract

By their very nature, great advances in immunology are usually underpinned by experiments carried out in animal models and inbred lines of mice. Also, their corresponding knock-out or knock-in derivatives have been the most commonly used animal systems in immunological studies. With much credit to their usefulness, laboratory mice will never provide all the answers to fully understand immunological processes. Large animal models offer unique biological and experimental advantages that have been and continue to be of great value to the understanding of biological and immunological processes. From the identification of B cells to the realization that γδ T cells can function as professional antigen presenting cells, farm animals have contributed significantly to a better understanding of immunity.

## Introduction

Great advances in immunology are usually supported by experiments carried out in animal models and by far, inbred lines of mice and their corresponding knock-out or knock-in derivatives, are the most commonly used animal systems in immunological studies. Though with much credit to their usefulness, laboratory mice will never provide all the answers to fully understand immunological processes. Also, some answers provided in mouse models are not applicable to other species of animals or humans. Large animal models offer unique biological and experimental advantages that have been and continue to be of great value to the understanding of biological and immunological processes.

The humble cow, the underestimated pig and the unassuming chicken have greatly influenced our current understanding of human immunology. For most immunologists dedicated to fundamental and applied research, it is easy to forget that B cells were first identified in chickens and vaccination first occurred because of a cow. Although there are far too many important events to discuss in this paper, we have chosen to highlight a few of the most important contributions of farm animals to the current understanding of immunology (Table 1).

**Table 1:** Selected major contributions of farm animals to immunology.

**Species**	**Contribution**
Cows	•Cowpox from udders to vaccinate humans •First recombinant subunit vaccine-FMDV VP1 •DEC205 identified as marker for DCs •Bovine RSV and TB as natural models for human disease
Chickens	•Immunization of chickens with *Pasteurella* to coin the term “vaccine” •Identification of cells from the *Bursa of Fabricius* (B cells) as antibody producing cells •First interferon identified and characterized •Marek's disease as natural model for human T cell lymphomas
Pigs	•Swine plague vaccine used during first global vaccination campaign in history •Xenotransplantation and fundamental mechanisms of tissue allorejection •Swine influenza and Nipah virus infections as natural models for human disease
Horses	•Serum from immune horses used to define the role of antibodies in protection against diphtheria
Sheep	•Identification that B cells are activated in lymph nodes •Somatic cell nuclear transfer-Dolly the sheep

## History of Vaccination

Edward Jenner published in 1798 a booklet entitled “*An Inquiry into the Causes and Effects of the Variolae Vaccinae, a disease discovered in some of the western counties of England, particularly Gloucestershire and known by the name of Cow Pox*” (1, 2) and although strictly speaking Jenner did not discover vaccination, he was the first person to use scientific rigor to prove protection from disease through targeted intervention. The English dairy farmer Benjamin Jesty (1737–1816) was the first person known to vaccinate against smallpox (3) protecting his family against the virus even after numerous exposures (3).

However, the idea and indeed the term *vaccination*, only came into the light spot 100 years later thanks to Louis Pasteur. This time the chicken takes a privileged position and the story was beautifully explained by Pasteur himself (4, 5) and has been romanticized in Paul De Kruif's book “Microbe Hunters” (6). In 1878 Pasteur inoculated chickens with “stale” cultures of *Pasteurella multocida*. The chickens became sick but recovered so he decided to re-inoculate them with a *fresh* culture. The chickens that had received the “stale” culture recovered whereas chickens that had not been pre-exposed to the stale cultures died. Pasteur recognized the similarities between his studies in chickens and what Jenner had published with smallpox. He coined the term “vaccine” (4, 5, 7) in honor of Jenner.

By the early 1880s, William Smith Greenfield in the UK (8, 9) and Pasteur working with Henri Thullier, Charles Chamberland and Pierre Paul Émile Roux in France (10, 11) had begun developing and testing vaccines against anthrax in sheep and cattle. A decade later, the German scientists Friedrich Loffler and Paul Frosch identified the first ever filterable infectious agent in mammals: foot and mouth disease virus (FMDV) and developed a fully protective heat-inactivated vaccine against it (12, 13); however an effective long-lasting and broadly protective vaccine against FMDV remains elusive.

Pigs also played an important role in early vaccinology studies. By the late 1800s swine plague or hog cholera (later discovered to be caused by a virus now called classical swine fever virus, CSFV (14) was killing hundreds of thousands of pigs across the word and was particularly of concern to the US pig producing industry, causing an impressive US$15 million a year in losses in 1875 (15) and US$20 million by 1878^1^. Once again, Pasteur and Thullier developed a vaccine to what is now thought to be the first ever vaccine against a viral infectious disease (16) and the first mass-vaccination campaign in history. In addition, it is rarely recognized that CSFV was the first animal virus ever to be cultured *in vitro* (17) and the techniques developed by Carl Tenbroek continue to be used today.

Horses have also contributed to the understanding of fundamental immunological mechanisms. In a series of experiments, Emile Roux working with Alexandre Yersin and followed by Emil von Behring and Shibasaburo Kitasato immunized horses to produce an “antidote” or immune sera against the diphtheria toxin that was eventually used to treat humans, an important step in understanding antibodies and humoral immunity (18). Behring won the Nobel Prize for Medicine in 1901 for this work.

Another milestone in vaccine development was the generation in the 1970's of vaccines to control Marek's disease (MD), a naturally occurring neoplastic disease in chickens caused by an oncogenic herpesvirus (19). MD vaccines are the first examples of the use of vaccination to protect against cancer (20, 21).

With the discovery of molecular biology techniques in the 1960's and 70's, the race was on to develop recombinant vaccines against numerous infectious diseases. The first report of a biosynthesized polypeptide vaccine was published in 1981 (22). The structural protein VP3 of FMDV was cloned and expressed in *E. coli* and the purified protein used to vaccinate six cattle and two swine, which developed neutralizing antibodies and were protected against challenge with FMDV (22). And new technologies have only helped to highlight the importance of farm animals in vaccine development: using a computational approach to assess protein-protein stability, Kotecha and colleagues (23) used molecular dynamic ranking to predict FMDV capsid stabilities and produced stabilized FMDV capsids based on these predictions, assessed their stability using X-ray crystallography and demonstrated their improved immunogenicity *in vivo* by vaccinating cattle. This demonstrates the potential value of structure-based design of vaccines to develop stabilized vaccine antigens for animals and humans alike.

## Innate Immunity

Although the innate immune system of animals is largely conserved, there are significant variations in the Pattern-Recognition-Receptor (PRR) structures of various species (24). It has been suggested that laboratory mice have not been subjected to the selective pressures that other animals have and so innate immune studies carried out in laboratory animals do not accurately inform human biology (25). It has been demonstrated that human and farm animal PRR responses to their ligands (24, 26) are more similar to each other than human-mouse PRR responses (26–28). Because PRR recognition is associated with adaptive immunity, a better understanding of these molecules in farm animals is likely to better inform on their effect in these animals as well as humans.

A major contribution of the chicken to fundamental innate immunity was the description in 1957 of the first interferon. Chicken embryos were exposed to influenza virus by Alick Issacs and Jean Lindenmann (29) and they identified an immune soluble element responsible for regulating virus infection. This discovery was certainly one of the scientific landmarks in cell biology in the Twentieth century and one which opened the doors of what we now know as innate immunity.

## B Cells

Perhaps the most recognizable contribution of the chicken to science, and immunology in particular, was in the definition of the two elements of adaptive immunity: the B-dependent and the T-dependent immunity.

The avian bursa of Fabricius, named after Hieronymus Fabricius of Aquapendente (30) is a sac-like structure located in the cloacal passage of the bird and its function remained elusive until well into the Twentieth century. Bruce Glick and Timothy Chang working at the Poultry Science Department at Ohio State University (30, 31) described how following the surgical removal of the bursa, chickens injected with *Salmonella typhimurium* “O” antigen failed to develop bacteria-specific antibodies. Glick and Chang wrote a paper entitled: “*The role of the bursa of Fabricius in antibody production*” and was rejected by leading scientific journals (30) and eventually published in *Poultry Science* (32). Several years later, the bone marrow in mammals was shown to be the equivalent of the bursa in birds (33), and so the term “B-lymphocyte” originated from “bursa-derived lymphocyte”. Several years later, Cooper et al. published a fundamental paper on the demarcation of the thymic and bursal and systems in birds and proposed the existence of equivalent systems in mammals (34).

The cannulation of lymphatic vessels was developed in the early Twentieth century in rats to study the lymphatic system but due to the complexity of the surgical procedure, sheep and then cattle were used extensively in the 1960s and 70s in lymphatic cannulation studies (35). In a series of adoptive transfers of lymph-migrating cells and fluid, Hall and colleagues first identified in sheep that antibody-secreting cells (ACS) encounter antigens and are activated in the lymph nodes (36), then migrate via the efferent lymphatics to the circulatory system, and that the immune response depends on an intact lymphatic system.

Cattle have also contributed to fundamental B cell immunology and the generation of a highly diverse antibody repertoire. Most vertebrates encode a large number of variable (V), diversity (D), and joining (J) gene segments and antibody diversity is achieved by recombination of these 3 segments. In contrast, cattle only express a limited number of V genes and so it is thought that antibody diversity is achieved recombination events and endogenous mutation mechanisms in the CDR3 region (37). Another unusual feature of bovine antibodies is their exceptionally long CDR3 regions (38). These long CDR3 and unusual mutation mechanisms result in “microfolds” within the CDR3 region that allow bovine antibodies to bind antigens that would normally be inaccessible (38).

A recent report demonstrated that cows can be immunized with a single HIV Env trimer and this results in potent HIV-specific nAbs which are dependent on the length of the CDR3 loops of bovine Ig (39). It has been suggested that this could be an efficient way of producing super-antibodies against other human pathogens. Transchromosomal cows have been engineered to produce large amounts of full human IgG molecules with pathogen specificity: MERS-CoV (40), Hanta virus (41), VEEV (42), and Ebola virus (43). This technology has the potential to generate prophylactic antibodies against emerging viral diseases.

On the other hand, chickens have serum IgM and IgA both of which are homologs of their mammalian counterparts; in addition, they express IgY, not found in mammals but thought to be an evolutionary ancestor for mammalian IgG and IgE. Chickens however do not have either IgE nor IgD but instead use a distinct process for generating antibody diversity that is distinctly different to mammals (44).

Engineers frequently look to nature for inspiration. Antibody engineers are no exception, modeling new therapeutics on molecules found in animals such as camels and cows. Indeed, 10% of bovine antibodies have unusually long heavy-chain CDR3s as part of their antigen-recognition sites. Stanfield et al. (45) have solved crystal structures of three new bovine Fab fragments and analyzed the five known structures to show that their ultra-long CDR H3s all adopt similar architectures composed of a knob domain containing a small conserved β-sheet connected by diverse disulfide-bonded loops that is separated from the antibody surface by a long conserved stalk. They propose that varying the length of the stalk and the positions and number of disulphide links in the knob may help drive antibody diversity. These structural insights could be leveraged to tailor antibody-based therapeutics.

In contrast to all other mammals, camelids (dromedaries, camels, llamas, etc) also have an unique antibody type similar IgG but with identical heavy chains lacking the CH1 domain and which do not pair with their corresponding light chains. These “heavy-chain antibodies” (HCAbs) display antigen-specific variable domains or “VHH” which are structurally and functionally similar to an IgG Fv but have only three CDR loops defining the antigen biding sites. Camel VHH domains, also called “nanobodies,” have been of great interest because of their stability and small size and strong affinity to their corresponding antigens. In fact, several camel VHH domain antibodies are in early preclinical development in oncology, infectious, inflammatory, and neurodegenerative diseases (44), the most recent example being the generation of broadly neutralizing antibodies to influenza in llamas (46).

## T Cells

Cytotoxic and helper T cells are generally considered to be phenotypically different due to the mutually exclusive expression of the co-receptors CD8αβ and CD4 and differences in MHC-restriction (class I vs. class II). However, between 3 (in normal individuals) and 60% (in certain pathologies) of human peripheral blood lymphocytes have been shown to be CD4/CD8 double positive (DP) T cells (47). Thymic and extra-thymic development of T cells has been studied mainly in mice and because the expression of CD8 and CD4 in mouse T cells for the most part mutually exclusive, CD4/CD8 DP lymphocytes have generally been ignored. Nevertheless, the presence of CD4/CD8 DP T cells in many animals makes it impossible to ignore these cells. Studies in pigs have shown that CD4/CD8 DP are a distinct subset of activated and/or memory T helper cells (48) and in humans the increase in circulating CD4/CD8 DP T cell frequency has been identified in autoimmune and chronic inflammatory diseases (49–53) suggesting the importance of this particular T cell population in human health.

Most circulating T cells in humans and mice are conventional T cells expressing the αβ T cell receptor (TCR) and either CD4 or CD8. Unlike mice, other species like cattle, pigs and chickens possess a substantial proportion of T cells expressing the γδ TCR cells in the circulation suggesting that circulating γδ TCR T cells have a more important role immunity than previously thought (54).

The fact that the phenotype and frequency of circulating and tissue resident T cells is so vastly inconsistent in different species suggests that immune responses to (vaccine) antigens are also distinct. It is assumed that that all animal species have a similar immune response to a particular antigen, but this is a statement to be reviewed in light of each host particularities. In addition, the TH1/TH2 polarization of T cells observed in response to particular antigens is a phenomenon of certain strains of laboratory mice and not of outbred mammals including farm animals and humans (55, 56). In fact, it has been shown that cytokine profiles defining TH1/TH2 responses to antigens in cattle are more similar to human responses than those observed in mice (57).

## Dendritic Cells

Dendritic cells (DC) as such, and their role in immunity were first described in the 1970s and in 1995 Ralph Steinman published a series of papers describing that a cellular receptor called “DEC-205” (now CD205) was expressed on mouse DC, was involved in antigen processing (58, 59) and was detected by the monoclonal antibody NLDC-145. In fact, it was 2 years earlier in 1993, that Chris Howard, a bovine immunologist working at the then called “Institute for Animal Heath” in the UK published a series of papers identifying an important and until then uncharacterized marker expressed on pseudo-afferent lymph veiled cells (also called ALDC) detected by the monoclonal antibody WC9 (now CC98) (60–63). Although Steinman's identification of mouse CD205 helped characterize the binding of CC98 to bovine CD205 (64), the importance of CD205 in identifying DC was first evident in cattle.

As mentioned above, Steinman's seminal work in characterizing DC using the mouse system has been one of the most important developments in cellular immunology of the Twentieth century, and one which lead to his Nobel laureate. However, the idea that a component of the immune system was involved in antigen processing and presentation had been proposed many times before. As mentioned above, cannulation of the lymphatic vessels is more practical in large than small animals, and this technique has been used to investigate DC biology. Afferent or peripheral lymph DC were first described in sheep in 1972 (65) as “very phagocytic dendritic macrophages that are involved in long term immunological reactions” that are very potent antigen presenting non-lymphoid cells (66) and that their phagocytic and antigen presentation capacities differed from “classical” peritoneal macrophages (67), therefore indicating that DC and macrophages were different cell types (67) several years before Steinman's observations (68). In addition, lymphatic cannulation of sheep has revealed important ontologic, phenotypic and functional characteristics of DC subsets that are relevant in other mammals, particularly humans (69, 70).

Similitudes and differences between swine and human DC/macrophage populations have recently been described (71). In one striking example and in contrast to studies performed in mice, swine and human cDC2, which are associated with Th2 responses, both express FcεRIα and are localized in or next to the tracheal and bronchial epithelia. These observations have been proposed to imply that swine and humans have similar allergen responses as opposed to mice. This theory is supported by the fact that localization of cDC2 helps them access antigens such as airborne allergens, and FcεRIα expression on these cells might help proliferation observed in allergic responses.

## γδ T Cells

As mentioned before and unlike mice, horses and humans, most other animals have a large γδ T cell compartment. For example, up to 70% of all blood lymphocytes in young calves are T cell expressing the γδ T cell receptor (TCR). Although the reasons for the enlarged T cell compartment in cattle, pigs and chickens is still unknown, their large numbers and ease of collection has resulted in great advances in γδ T cell biology knowledge not only for farm animals, but also for humans. For example, APCs were shown to influence γδ T cell proliferation (72, 73). Cynthia Baldwin's lab has defined antigen-specific bovine γδ T cell responses in various systems (74–76) and Adrian Smith's lab has done similar observations in chickens (77, 78). It has also been shown that bovine γδ T are potent regulatory T cells (79), an observation that is also true for a subset of human γδ T cells (80, 81). These results in farm animals have and continue to enhance our understanding of human γδ T biology (82). Perhaps the most important one was the realization that a subset of bovine γδ T cells expressed MHC class II and co-stimulatory molecules on their surface, a characteristic normally attributed to macrophages, B cells and DC but not T cells (83). Bovine γδ T cells were also shown to phagocyte antigens and of MHC II-restricted presentation to CD4+ T cells (83). This function of bovine γδ T cells was subsequently reported in pigs (84) and much later in mouse (85) and human (86–88) γδ T cells.

## Somatic Cell Nuclear Transfer

Perhaps the best known contribution of any farm animal to scientific progress was the somatic cell nuclear transfer that gave origin to Dolly, the sheep (89). Although nuclear transfer itself is not a direct contribution to immunology, nuclear transfer technology has directly influenced many immunological concepts underpinned by technologies such as induced pluripotent stem (iPS) cells and CRISPR-Cas systems.

Clustered regularly interspaced short palindromic repeats (CRISPR) is a RNA-guided endonuclease used both *in vivo* and *in vitro*. Genetically modified animals becoming more common and their availability can be exploited in many applications such as comparative immunology, physiology and disease, to generate *in vivo* bioreactors to produce complex proteins, or to produce genetically modified organs for transplantation in humans (90).

## Animal Models of Infection and Disease

Although the majority of pharmaceutical research is performed in laboratory mice models, it is clear that humans are not “large mice.” By a large extent, studies in laboratory mice have been the victim of over interpretation; for example, by extrapolating successful pre-treatment in mice to therapeutic treatment in men. The weakness of the mouse model in pharmaceutical research was recently highlighted in a study showing that inflammatory responses in mouse models do not correlate with human inflammatory disease (91). An additional study showed a close similarity in expression profiles of immune-related genes between humans and pigs (92).

Cattle, pigs, and chickens, are useful, valid, and valuable models to study human infectious diseases and important clinical targets in their own right. Both humans and cattle are the natural hosts for tuberculosis (being infected with the genetically-related *Mycobacterium bovis* and *Mycobacterium tuberculosis*, respectively) and the bovine and human diseases share many similarities in terms of immunity and pathology (93), whereas the mouse model of tuberculosis does not provide a faithful representation of the disease in humans (94). Similarly, bovine respiratory syncytial virus (bRSV) is closely related to human (h) RSV and the pulmonary pathology, immune responses and epidemiology seen in young calves and children are very similar (95). Swine have been shown to be a more faithful model for human influenza infection and immunity studies and the same strains of influenza infect both humans and pigs because the distribution of influenza virus receptors and physiopathology are similar in both species. The transfer of maternal-derived antibodies (MDA) to new born pigs enables fancy vaccine study design to elucidate the role of MDA in immunity (96, 97), vaccine efficacy and in enhancement of respiratory disease (98).

Gnotobiotic piglets have been used to study various human gastrointestinal pathogens. For example, human noroviruses are antigenically and genetically related to swine noroviruses and unlike mice, humans and pigs show genetic susceptibilities to noroviruses depending on their histoblood group antigen phenotypes and the virus strain. Similarly, gnotobiotic pigs have been used in rotavirus research to study disease pathogenesis and identify virus-specific IgA and ASC as correlates for protection and vaccine efficacy in children (99). Pigs have also been proposed to be better models than mice for many other infectious diseases including female genital infection with *Chlamydia trachomatis, Helycobacter pylori, Neisseria meningitides*, and Nipah virus among others because of the natural susceptibility of pigs to these pathogens (100).

Endogenous retroviruses were first discovered in pig kidney cell lines in 1971 (101) and are now known to be present in most, if not all, mammalians. The presence and potential reactivation of endogenous retroviruses has very important consequences in both allo- and xeno-transplantation.

Immunotherapy is becoming more popular in clinical trials and vaccine efficacy studies. The success of immune cell therapies partially depends on the effective delivery of cells to target organs, a process that invariably involves the lymphatic system. DC migration in mice has not proven to be very informative, however, DC migration in pigs may be able to answer several question on DC migration that cannot be addressed otherwise. These studies demonstrate that using large animals to investigate immune cell trafficking will help improve immunotherapies in humans (102).

## Xenotransplantation

In 1906 the French surgeon Mathieu Jaboulay (1860–1913) implanted a pig's kidney into one woman and a goat's liver into another thus starting the idea of xenotransplantation; unsurprisingly, both women died (103). The acceptance or rejection of a donor's organ or cells is fundamentally an immunological event. Cellular rejection involves NK and T cells that recognize foreign antigens on the grafted tissue. Using xenotransplantation models (pig-to-rat, pig-to-primate, and pig-to-human), the main mechanism for organ and tissue rejection has been proposed to involve arteriosclerosis, or thickening of the arterial walls. This process if thought to be caused by activated and allo-reactive lymphocytes that migrate over time to the transplanted organ (104). Arteriosclerosis is a major cause of chronic organ rejection (103).

## Developmental Immunology

Studies in laboratory mice have underpinned many concepts of immune tolerance and the generation of immune responses in the neonate. However, the peripheral immune system in mice remains unpopulated during pregnancy and it is only after birth that B and T cells begin to emmigrate to the periphery. In contrast, lymphoid cells circulate through the fetus in humans and large animals well-before birth and specialized lymphoid tissues are also well-developed and populated by the time of birth and are able to respond to a number of antigens (105, 106). Certainly the immune system in neonate humans and large animals is not matured, but calves, lambs and piglets can be more useful than mice in understanding immune responses during pregnancy and in new borns and these studies can be used to better inform human developmental immunology. This advantage over mice has recently been used to develop extracorporeal support technologies using neonatal lambs with the ultimate objective to use these technologies in premature children (107).

## Large Animal Models in Vaccinology

Perhaps one of the most common uses of large animal models is in the development of vaccines with several advantages over mice. The serial collection of peripheral blood from animals such as pigs, cattle, chickens, and horses allows for immunokinetic studies to be possible in response to vaccination or infection at the level of the individual. These immunokinetic studies can be used to correlate immune responses generated with protection after challenge with the relevant pathogen. In vaccinology studies using mice, the typical approach would be to sacrifice groups of mice sequentially and harvest spleen and blood, so the immune response to vaccination at the individual level is not normally achieved.

Large animals also provide several advantages over mice when investigating mucosal immunity. When mice are vaccinated or inoculated intranasally, it is common for the inoculum to be digested because anesthetized mice can both swallow and inhale the material placed on their nose. In addition, the structure of the mucosal associated lymphoid tissue (MALT) differs significantly in mice from that of large animals and humans; for example mice do not have tonsils but instead have undefined networks of MALT, whereas cattle, pigs and sheep have well-defined tonsils (108–110). In the case of vaccine delivery through the skin, cattle, and pigs appear to be better suited than mice for these studies. Skin thickness, structure of the epidermis and the presence and distribution of Langerhan's cells are among many characteristics that humans and cattle and pigs have in common and which are practically relevant in transcutaneous immunizations (111).

## Limitations of Animal Models

The process of selecting a relevant and appropriate animal model is a balanced and complicated exercise due to the diversity in vertebrate physiology, adaptive and innate immunity. Studies in mice, for example, have shown the efficacy of vaccines against FMDV, however these efficacy studies have failed to be translated to the target species (cattle and pigs), presumably due to fundamental differences in the immune systems of model organisms and target species and the ability of the virus to mutate in these animals (112). It has recently been shown that because immunoglobulin subclass diversification occurred after speciation (113, 114) a particular immunoglobulin subclass in one species bears no functional homology to one of the same name of another species (115). Thus, our knowledge of the functions of IgG1 in mice cannot be extrapolated to other mammals. Characterizing generating reagents for each animal model hinders the development and usefulness of any of these models and therefore limiting the usefulness of cows, cattle or chickens as models for human immunology.

Mice and rats are and will probably continue to be the chosen model organisms over farm animals. Mice can be readily mutated (knock in or knock out) to study immunological pathways; so far this has been proven to be very difficult—and expensive—in large animals. As mentioned above, the availability of reagents to study immune cells and processes in mice far out competes the availability of these reagents for large animals. Pharmacokinetic and toxicology studies would be prohibitively expensive in pigs, horses or cattle, so small rodents and rabbits are the best organisms to use in these studies. In addition, studies in mice have been fundamental in the discovery of antibiotics, chemotherapy agents and more recently CAR-T cell therapies that can be directly applied to humans. Genetic homogeneity, low cost, the availability of biologically-relevant mutants and reagents make the mouse the optimal animal model for many academic and industrial researchers.

## Conclusions

Farm animals have historically contributed and continue to contribute to fundamental and applied immunology. The use of these animals in research is not difficult as long as the appropriate facilities and reagents are available. Dedicated housing, cost, biosecurity, and genetic variability are some of the many disadvantages confronted when using farm animals in research. However, selecting an appropriate animal model should be more than just a matter of accessibility and common practice (116) but should be based on the scientific question to be addressed and its relevance.

## Author Contributions

All authors listed have made a substantial, direct and intellectual contribution to the work, and approved it for publication.

### Conflict of Interest Statement

The authors declare that the research was conducted in the absence of any commercial or financial relationships that could be construed as a potential conflict of interest.

## Footnotes

^1^(1881). Swine Plague. *Science* 2, 121
